# Cognitive, psychological, and physiological effects of a web-based mindfulness intervention in older adults during the COVID-19 pandemic: an open study

**DOI:** 10.1186/s12877-024-04766-z

**Published:** 2024-02-14

**Authors:** Samantha Galluzzi, Mariangela Lanfredi, Davide Vito Moretti, Roberta Rossi, Serena Meloni, Evita Tomasoni, Giovanni B. Frisoni, Alberto Chiesa, Michela Pievani

**Affiliations:** 1 Laboratory Alzheimer’s Neuroimaging and Epidemiology, IRCCS Istituto Centro San Giovanni Di Dio Fatebenefratelli, Brescia, Italy; 2 Unit of Psychiatry, IRCCS Istituto Centro San Giovanni Di Dio Fatebenefratelli, Brescia, Italy; 3 Alzheimer’s Rehabilitation Unit, IRCCS Istituto Centro San Giovanni Di Dio Fatebenefratelli, Brescia, Italy; 4 University Hospitals and University of Geneva, Geneva, Switzerland; 5 Istituto Mente E Corpo, Bologna, Italy; 6 Associazione Di Psicologia Cognitiva - Scuola Di Psicoterapia Cognitiva, Rome, Italy

**Keywords:** Older adults, Mindfulness, Web videoconference, Cognitive, Psychological, EEG

## Abstract

**Background:**

The development of effective strategies to maintain good mental health of older adults is a public health priority. Mindfulness-based interventions have the potential to improve psychological well-being and cognitive functions of older adults, but little is known about the effect of such interventions when delivered through internet. During the COVID-19 pandemic we evaluated short- and long-term cognitive, psychological, and physiological effects of a mindfulness-based intervention (MBI) delivered via web-based videoconference in healthy older adults.

**Methods:**

Fifty older adults participated in an 8-week MBI, which comprised structured 2-h weekly group sessions. A comprehensive evaluation encompassing cognitive (verbal memory, attention and processing speed, executive functions) and psychological assessments (depression and anxiety symptoms, mindfulness, worries, emotion regulation strategies, well-being, interoceptive awareness and sleep) was conducted. Additionally, electroencephalography (EEG) data were recorded before and after the MBI and at the 6-month follow-up (T6). Data were analyzed using an intention-to-treat approach, using linear mixed models adjusted for age. The effect size for time was computed as omega squared.

**Results:**

We observed significant improvements from pre-MBI to post-MBI and at the T6 across several measures. These improvements were notable in the areas of verbal memory (California Verbal Learning Test, *p* ≤ .007), attention and executive functions (Trail Making Test A and BA, *p* < .050), interoceptive awareness (Multidimensional Assessment of Interoceptive Awareness, *p* = .0002 for self-regulation and *p* < .05 for noticing, body listening, and trusting dimensions), and rumination (Heidelberg Form for Emotion Regulation Strategies, *p* = .018). These changes were associated with low to medium effect size. Moreover, we observed significant changes in EEG patterns, with a decrease in alpha1 (*p* = .004) and an increase in alpha2 (*p* < .0001) from pre-MBI to T6. Notably, improvements in TMTBA and rumination were correlated with the decrease in alpha1 (*p* < .050), while improvements in TMTA were linked to the increase in alpha2 (*p* = .025).

**Conclusions:**

The results of our study show that a web-based MBI in older adults leads to improvements in cognitive and psychological measures, with associated modulations in specific brain rhythms. While these findings are promising, further controlled studies are required to validate these preliminary results.

**Trial registration:**

The trial has been registered with the United States National Library of Medicine at the National Institutes of Health Registry of Clinical Trials under the code NCT05941143 on July 12, 2023.

**Supplementary Information:**

The online version contains supplementary material available at 10.1186/s12877-024-04766-z.

## Background

The global population of older adults is poised to experience a significant surge in the coming years. It is estimated that between 2015 and 2050, the proportion of the world's population aged 60 and above will rising from 12 to 22%. In absolute terms, this equates to an expected increase from 900 million to 2 billion individuals [1]. Consequently, the promotion of the healthy aging has emerged as a critical public health imperative. Mental health constitutes an integral component of healthy aging, encompassing a multifaceted state of psychological and cognitive well-being that empowers older adults to sustain their everyday functioning. In recent years, mindfulness-based interventions (MBIs) have gained recognition as a viable strategy for enhancing mental health across diverse populations [2].

From a psychological perspective, mindfulness is commonly defined as the practice of wholeheartedly focusing on the present moment while maintaining a non-judgmental awareness of both inner and outer experiences [3]. Older adults have demonstrated higher self-reported mindfulness, greater emotional resilience, and day-to day emotional well-being in comparison to their younger counterparts [4, 5]. This increased level of mindfulness in older adults has a positive effect on their mental health [6]. However, it is worth noting that older adults also face a higher risk of encountering stressful life events such as bereavement or the onset of chronic illnesses compared to younger adults [7]. Consequently, protective factors cultivated through mindfulness-based interventions (MBIs) can act as buffers against these stressors, offering an effective means of enhancing mental health in older adults [8]. Accordingly, a growing body of research suggests that MBIs in the elderly can have beneficial effects on psychological outcomes, including emotional well-being, stress, depression and anxiety, rumination and worry [4, 9–13]. Moreover, there is evidence of improvements in cognition, particularly attention and executive functions [14, 15].

The COVID-19 pandemic has catalyzed a significant shift in the delivery of mental health care toward digital information and communication technologies [16]. This transformation has spurred a heightened demand for online interventions. The value of internet-based delivery has been underscored, even in the context of MBIs [17], and their beneficial effects have been demonstrated during the COVID-19 crisis [18–20]. This digital approach offers several advantages, including cost effectiveness, scalability and enhanced accessibility. Online MBIs can be administered in various formats, either synchronously through virtual classrooms or asynchronously through web-based courses or smartphone applications. However, it is important to note that while the use of mindfulness apps may hold promise [21], the majority of them have not undergone testing in randomized clinical trials, and concerns related to privacy protection and security may arise [22].

Concerning efficacy, the most recent and large meta-analysis of randomized controlled trials, which evaluated the effectiveness of online MBIs in improving mental health in clinical and non-clinical samples, showed small to moderate beneficial effect on depression, anxiety, stress and mindfulness [23]. Although most of MBIs were delivered through self-help web-based courses, subgroup analyses demonstrated significantly higher effect sizes for stress in guided compared to unguided interventions, suggesting the importance of teacher involvement to learn and understand mindfulness practice [23]. This finding was in line with previous studies showing higher efficacy of online MBIs when delivered through videoconference than on website [24, 25].

Additionally, it is essential to recognize the significance of gathering physiological data alongside clinical outcomes in studies of MBIs. The measurement of biomarkers offers valuable insights into the physiological mechanisms that underlie the effects of an intervention, thereby supporting a comprehensive understanding of its clinical effectiveness [26]. Furthermore, the collection of biomarker data can facilitate the linkage of physiological and clinical pathways to an individual’s health and well-being. This, in turn, can contribute to the development of personalized interventions that are tailored to an individual's specific needs and characteristics. As a result, an increasing number of studies have examined the neurobiological effects of MBIs in older adults, exploring parameters such as brain structure and function [27, 28], as well as brain activity [29, 30]. Nevertheless, only a minority of these studies have explored correlations between clinical and biological outcomes, thereby limiting their potential contribution to our understanding of clinical-physiological pathways.

In this study, we incorporated physiological data alongside clinical information, utilizing electroencephalography (EEG), due to its straightforward, cost-effective, and non-invasive nature for measuring neural activity in the brain. The dominant oscillatory pattern in EEG recordings is the alpha activity, which plays a foundational role in processes related to attention and consciousness [31]. A review of studies investigating EEG patterns associated with mindfulness practice revealed a consistent pattern of changes, with increased alpha and theta power being most frequently reported. These changes may indicate an enhancement in attentional capacity and a reduction in overall arousal levels [32]. The variation in findings across studies has been attributed to a lack of standardization in EEG signal processing techniques and of their associated parameters [32].

There are notable research gaps in the field of online MBIs that require further investigation. Firstly, although evidence showed high levels of digital competence and feasibility of digital technology for health promotion in older adults [33], the efficacy of online MBIs in older adults remains largely unexplored—only two out of 97 studies included in the aforementioned meta-analysis [23] involved this population. Secondly, the same meta-analysis evidenced that online MBIs have primarily focused on psychological outcomes, with a relative neglect of cognitive functions [23]. This is in contrast to traditional mindfulness-based programs, which have extensively studied cognition, such as attention and executive functions [15]. Thirdly, there is a dearth of information regarding long-term effects of online MBIs. The existing studies typically have relatively short follow-up periods of 1–3 months [23], making it uncertain whether the positive effects of online MBIs can be sustained over an extended period. Lastly, to address the lack of standardization in EEG analysis, methods that may provide more precise estimates of modulations in alpha activity, like the Individual Alpha Frequency, have been proposed [34]. Additionally, exploring of narrow frequency bands within the alpha range, such as alpha1 and alpha2, is valuable as they are associated with distinct cognitive processes [35]. Investigating these specific frequency bands can contribute significant insights into the neural underpinnings of MBIs.

Hence, the objective of this study is to assess both short and long-term cognitive, psychological, and physiological outcomes of an adapted 8-week MBI delivered through live web-based videoconferencing among a group of healthy older adults. This intervention was conducted during the initial year of the COVID-19 pandemic, at a time when Italy was emerging from its first lockdown, although certain restrictions remained in effect.

## Methods

### Participants

From September 2020 to March 2021, we consecutively recruited fifty older adult who were new to mindfulness practice. These participants were part of a larger cohort of 70 older adults, with the initial 20 individuals being enrolled in September 2019 and exposed to a traditional, in-person MBI. The in-person group sessions were halted in February 2020 in response to the first COVID-19 lockdown. In response to this situation, we made adjustments to the study protocol by transitioning the MBI format from in-person sessions to live web-based videoconferencing.

The study protocol, as well as the subsequent amendment, received approval from the local Ethics Committee, specifically the Ethics Committee of the IRCCS Istituto Centro San Giovanni di Dio Fatebenefratelli. Participant recruitment was conducted within the community – with the majority of individuals being residents of the province of Brescia, an urban area characterized by medium to high socioeconomic status. Recruitment was carried out through various means, including internet newsletters, distribution of flyers, and promotional efforts on social media platforms like Facebook. Additionally, some participants were referred from a list of individuals who had previously participated in previous research studies and had consented to be contacted for future research endeavors.

Inclusion criteria encompassed the following: (i) older adults residing in the community, aged between 60 and 75 years; (ii) performance within the normal range on standardized cognitive tests, as further detailed in the section on cognitive assessments. Exclusion criteria were defined as follow: (i) the presence of clinically significant depression or anxiety; (ii) presence of current major neurological conditions (including stroke, dementia or cognitive impairment, tumor) or psychiatric disorders (including major depressive disorder, bipolar disorder, as well as drug and alcohol dependence). These conditions were evaluated in accordance with the diagnostic criteria of the International Statistical Classification of Diseases and Related Health Problem, 10th revision [36] and/or Diagnostic and Statistical Manual of Mental Disorders, 5th edition [37]; (iii) the presence of a serious chronic disease or an acute unstable illness, spanning various medical domains such as respiratory, cardiovascular, digestive, renal, metabolic, hematologic, endocrine, infectious, or malignancy, as defined by the International Classification of Diseases, 10th Revision [38]. These criteria were established to ensure that individuals with medical conditions that could impede their ability to complete or hinder accurate data collection were excluded. Additionally, individuals who were actively engaged in regular mindfulness practice at the time were also excluded from the study.

A total of 54 subjects were initially screened for the study. Among them, 4 individuals were excluded from participation. The reasons for exclusion were abnormal cognitive tests (*n* = 2) and clinically significant depression (*n* = 2).

## Procedures

### Study protocol

**Fig. 1:**
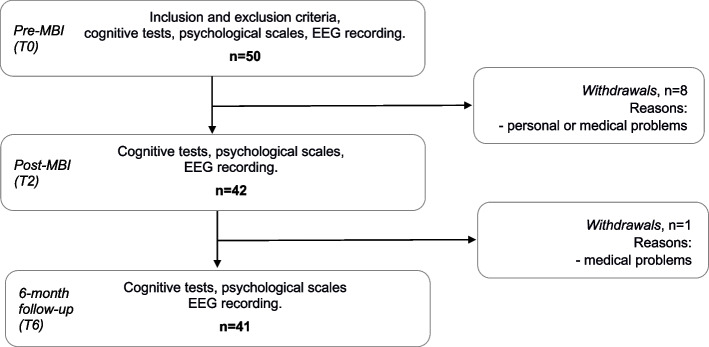
Study design and participant flow

This study was conducted an open trial, and it has been registered with the United States National Library of Medicine at the National Institutes of Health Registry of Clinical Trials under the code NCT05941143 as of 12/07/2023. Participants underwent assessments in three distinct in-person time-points (Fig. 1). The initial assessment occurred during the pre-MBI visit (T0), which took place within 15 days before the commencement of the MBI. This visit served to confirm eligibility in accordance with the previously outlined inclusion and exclusion criteria and to collect cognitive and psychological data. On a separate day, EEG data were recorded.

The post-MBI visit (T2) occurred within 15 days after the completion of the MBI program while the follow-up visit (T6) took place 6 months after the conclusion of the MBI, with a window of ± 2 weeks after the end of the MBI. These visits entailed the same procedures as the baseline assessment, encompassing the administration of cognitive tests, psychological scales and EEG recording.

### Intervention

The MBI used in this study was primarily based on an existing intervention known as the Mindfulness-Based Cognitive Approach for Seniors (MBCAS) proposed by Zellner Keller and colleagues for older individuals [39]. The MBCAS program draws inspiration from various established mindfulness programs, including Mindfulness-Based Stress Reduction [40], Mindfulness-Based Cognitive Therapy [41], and Mindfulness-Based Relapse Prevention [42]. The program was delivered through live videoconferences using the WebEx connection via the StarLeaf platform. Participants accessed the program by following a link provided by the intervention staff, and most frequently joined the sessions from their home computer.

#### MBI program

The core objectives of the program are to educate participants on transforming their relationship with negative thoughts and emotions by nurturing awareness and cultivating an accepting, non-judgmental, and compassionate attitude towards them. Additionally, the program aims to empower individuals to effectively address cognitive, physical, and psychological challenges commonly encountered by older people.

A detailed session-by-session description of the program was developed by the authors (M.L., S.G., A.C.) to adapt the MBCAS program content for the Italian context and standardize the intervention. In contrast to the extended 8-month format of MBCAS, the MBI program implemented in this study featured 8 weekly group sessions, each lasting 2 h. Participants learned three main practices. The body scan: this practice involves a gradual sweeping of attention through the entire body, starting from the feet and moving upwards to the head. Participants were instructed to focus noncritically on any sensation or feeling in different body regions, with periodic suggestions for breath awareness and relaxation; the sitting meditation, which involves mindful attention to the breath, the rising and falling of the abdomen, and other sensory perceptions. Participants were encouraged to cultivate a state of nonjudgmental awareness of their cognitions and of the stream of thoughts and distractions that continuously flow through the mind; finally, hatha yoga practice, which incorporates breathing exercises, simple stretches, and postures designed to strengthen and relax the musculoskeletal system (for additional details, refer to Supplementary Table 1).

MBI sessions also involve psycho-education on various topics. These topics included: the stress reaction cycle and the stress management, exploring the interconnected nature of emotions, thoughts, behaviors recognising and addressing patterns of cognitive and emotional reactivity that may contribute to distress, learning to identify and manage dysfunctional thought patterns, and promoting self-care practices.

#### MBI facilitator

The mindfulness program was led by a certified mindfulness facilitator (M.L.), who had completed programs in both Mindfulness Based Stress Reduction and Mindfulness-Based Cognitive Therapy. Although formal assessment of treatment fidelity was not carried out, the sessions were audio-recorded for reference. Additionally, the intervention facilitator held monthly meetings with a supervisor (A.C.) possessing extensive expertise in mindfulness practice and teaching. The facilitator made herself available outside of the regular sessions to offer participants additional support and guidance via phone.

#### Home practice

Participants were encouraged to incorporate the newly acquired mindfulness skills into their daily lives through regular home practice. They were advised to allocate approximately 45 min each day for mindfulness exercises. The home practice regimen included the following components: formal mindfulness practice (i.e., meditation and yoga) for about 30–35 min a day, and informal practice or about 10–15 min a day focused on bringing mindful awareness to everyday activities. To support and guide their home practice, participants were provided with MP3 audio recordings of the meditations introduced during the MBI sessions. Participants received weekly handouts that covered the topics discussed during the sessions. In addition to these handouts, they were provided with a workbook containing homework assignments and self-report home practice diary sheets. These materials were distributed via email to all participants, ensuring that even those who couldn't attend the sessions in person had access to them. Finally, a booster session was organized for participants two months after the completion of the 8-week MBI. This session lasted for 4 h and included group mindfulness practices and informative lectures (see Supplementary Table 1 for details).

#### MBI adherence

The study collected data on participant attendance at the weekly online sessions. Additionally, the reasons for any missed sessions were documented. The completion rate was calculated as the percentage of participants who successfully attended all eight sessions by the end of the 8-week MBI. Participants were asked to report the frequency and duration of their mindfulness practice at home. They were specifically requested to provide details about the number of days they practiced mindfulness and the average duration (in minutes) of their practice, for both formal and informal practices. This information was collected at the post-MBI visit (T2) and the 6-month follow-up (T6).

## Measures

### Clinical assessment

The Mini-International Neuropsychiatric Interview (MINI) [43] conducted by the study physician (S.G.), was used to assess the presence of psychiatric illnesses, in line with the exclusion criteria. Additionally, depressive and anxious symptoms were evaluated through the 30-item Geriatric Depression Scale and the State-Trait Anxiety Inventory, respectively.

The MINI was employed to exclude individuals with current clinically significant depression or anxiety, using predefined cut-off scores (≥ 11 for depressive symptoms [44] and ≥ 55 for anxiety symptoms [45]). Comorbidity was assessed with the Cumulative Illness Rating Scale [46], considering medical history and current medication use. This scale assessed disease severity across 14 organ systems, assigning ratings on a scale from 0 to 4, yielding a total score from 0 to 56. Additional evaluations included the Revised NEO Personality Inventory [47] for personality traits, the Cognitive Reserve Index questionnaire [48] to measure cognitive reserve and the Multifactorial Memory Questionnaire [49] to evaluate memory concerns. Sleep quality was assessed by using the Pittsburgh Sleep Quality Index [50].

### Cognitive tests

Cognitive evaluation encompassed a range of tests aimed at assessing different cognitive domains. These included tests for overall cognitive function (Mini Mental State examination [51]), verbal memory (California Verbal Learning Test, CVLT [52]), attention and attention switching (Attentional Matrices [53]; Stroop test, time [54]; Trail Making Test A, TMTA, Trail Making Test B, TMTB [55]), and executive functions (Trail Making Test BA, TMTBA; Stroop test, errors; Wisconsin Card Sorting test [56]). To account for individual differences in age and education, the scores were adjusted based on normative data from the Italian population. The CVLT, in particular, measures an individual's verbal episodic memory. The CVLT is a word-list task that involves orally presenting 16 words to participants and instructing them to recall as many words as they can. Subsequently, semantic cues provided to aid recall. The task includes immediate recall, short delay recall, and 30-min long-delay recall trials. To mitigate potential learning effects, parallel forms of the test were utilized. The TMT assesses attention switching and executive control. In the TMTA version, participants are required to connect consecutive numbers (TMTA), while in the TMTB version they must alternate between numbers and letters. Faster completion times indicate better attention abilities. Furthermore, the time difference between task A and B was calculated (TMTBA), representing the cognitive demand associated with task B and reflecting performance in concept shifting within the domain of executive function.

### Psychological scales

The psychological evaluation consisted of self-administered scales that assessed various aspects, including chronic worry (Penn State Worry Questionnaire [57]), psychological well-being (Warwick-Edinburgh Mental Well-being scale [58]), and emotion regulation strategies by the Emotion Regulation Questionnaire [59] and the Heidelberg Form for Emotion Regulation Strategies, HFERST [60]. The HFERST comprises 28 items designed to assess eight different emotion regulation strategies: rumination, reappraisal, acceptance, problem solving, suppression of emotional expression, suppression of emotional experience, avoidance and seeking social support. Participants rated these items on a 5-point Likert scale, with higher scores indicating a more frequent use of these strategies (1 = never; 5 = always). Mindfulness-related aspects were evaluated using the Five Facet Mindfulness Scale, which assesses dispositional mindfulness [61]. Additionally, the Multidimensional Assessment of Interoceptive Awareness (MAIA, [62]) was used to measure interoceptive awareness. The MAIA consists of 32 items and encompasses eight distinct subscales, which include: noticing body sensations, not distracting from negative sensations (e.g., painful), not worrying about uncomfortable sensations, sustaining attention to sensations, awareness of the connection between sensations and emotions, regulating psychological distress through attention to sensations, active listening to the body for insights, and experiencing one’s body as safe and trustworthy.

The Italian version of the clinical and psychological scales was employed, and validation studies indicated an acceptable internal consistency, with the majority of Cronbach’s alpha values falling between 0.60 and 0.80 (see Supplementary Table 2 for details).

## Data analysis

### EEG acquisition and data processing

The EEG recordings were conducted using the EBNeuro Galileo NT PMS 2.40-SPA device. Subsequently, the spectral analysis of the EEG data was performed with Brain Vision Analyzer 2.2 software. The analysis adhered to contemporary EEG guidelines [63] following a standardized protocol. Power density calculations for EEG were computed with a frequency resolution of 0.5 Hz, covering a range from 2 to 45 Hz. This analysis was computed by a digital power spectrum analysis, based on the Fourier Fast Transform method (FFT) using the Welch technique, Hanning windowing function, and with no phase shift. The power spectra were obtained by averaging data from all recording electrodes. In this analysis, the focus was set on two specific anchor frequencies: the theta/alpha transition frequency (TF) and the individual alpha frequency (IAF) peak [64]. As our EEG recordings were conducted during rest, the TF was determined, which serves as an estimate of the frequency where the theta and alpha spectra intersect. The TF was computed as the frequency corresponding to the minimum power value within the alpha frequency range. Subsequently, the power of the IAF frequency was determined, which corresponds to the power value at the higher peak within the extended alpha range (6–14 Hz). Based on the values of TF and IAF, the frequency band range for each subject was estimated as follows: the low alpha band (including alpha1 and alpha2) ranged from TF to IAF, while the high alpha band (or alpha3) extended from IAF to IAF + 2. The alpha1 and alpha2 bands were determined for each subject as follows: alpha1 ranged from TF to the midpoint of the TF-IAF range, and alpha2 extended from the midpoint to the IAF peak [60]. The mean frequency ranges computed for the entire group of subjects are as follows: alpha1, 6.9–8.9 Hz; alpha2, 8.9–10.9 Hz; alpha3, 10.9–12.9 Hz. Lastly, the relative power spectra were calculated for each subject within the individually determined frequency bands. This was achieved by computing the ratio between the absolute power and the mean power spectra across the frequency range of 2 to 45 Hz, resulting in the relative power density for each frequency band. Specifically, the relative band power for each band was determined as the mean of the relative band power for each frequency bin within that specific band.

### Statistical analysis

Statistical analysis was conducted using the Statistical Software SPSS version 26.0. Significance was set at *p* < 0.05, and no correction for multiple testing was applied due to the exploratory nature of the study. We employed an intention-to-treat approach and utilized a mixed-effects model for repeated measures in the analysis. Various models were performed for cognitive/psychological measures and EEG variables as the dependent variables, with time (pre-MBI, post-MBI, and T6) acting as the within-subject factor. Estimated mean changes from baseline and their standard errors were reported. Given that participants who withdrew from the study were significantly older than those who completed the study (74.3 ± 5.6 vs 69.0 ± 4.6, *p* = 0.004), age was included as a covariate in the mixed-effects models. No other sociodemographic or clinical characteristics showed significant differences between the two groups.

Given the limited informativeness of significance levels in small sample sizes, we calculated the effect size for time as omega squared (Ω^2^). This was determined using the relative F-statistic, degrees of freedom, and the sample size in R statistic. Effect sizes with Ω^2^ values less than 0.01 are considered very small, those between 0.01 and 0.059 are categorized as small, values between 0.06 and 0.139 are regarded as medium, and values equal to or greater than 0.14 are considered large effects [65].

We conducted correlations between cognitive/psychological measures and EEG variables that exhibited significant improvements in the mixed models. Specifically, we defined T2change as the difference between post- and pre-MBI values and T6change as the difference between T6 and pre-MBI values. To ensure that higher values consistently reflected improvement in both cognitive/psychological measures and EEG alpha modulation, we multiplied change scores by minus one for measures where lower values indicate better performance.

## Results

### Descriptive analysis

Table 1 provides an overview of the characteristics of the older adults who participated in the MBI (*n* = 50). The mean age of the participants was 69.9 ± 5.1 years. The majority of participants were female (74%) and they had a relatively high level of education, with an average of 14.2 ± 5.2 years of education. Their cognitive reserve, as measured by the Cognitive Reserve Index questionnaire, was medium to high, with a mean score of 126.5 ± 22.2. The Cumulative Illness Rating scale yielded a mean total score of 5.4 ± 3.9, indicating low comorbidity. In terms of cognitive functioning, participants scored within the normal range on general cognition, as measured by the Mini Mental State Examination, as well as in the other cognitive domains (Supplementary Table 3). The score on the Revised NEO Personality Inventory was within the average range in each dimension (Supplementary Table 3). Additionally, depression and anxiety symptoms were below the clinical cut-off scores used for the Geriatric Depression Scale and the State-Trait Anxiety Inventory. Detailed information on the psychological scales administered at baseline can be found in Supplementary Table 4 [see Supplementary Tables]. We observed in our participants scores consistent with higher levels of dispositional mindfulness and well-being, higher use of emotion regulation strategies, higher worries and lower interoceptive awareness relative to the Italian validation samples.

**Table 1:** Sociodemographic and clinical features, and adherence to mindfulness-based intervention (MBI) of older adults enrolled in the study

	***N* = 50**
***Sociodemographics***	
Age	69.9 ± 5.1
Gender (female)	37 (74%)
Education (years)	14.2 ± 5.2
***Physical and cognitive health***	
Cumulative Illness Rating Scale, total score	5.4 ± 3.9
Mini Mental State Examination	28.7 ± 1.1
Cognitive Reserve Index questionnaire, total score	126.5 ± 22.2
***Depression and anxiety symptoms***	
Geriatric Depression scale	5.2 ± 3.7
State-Trait Anxiety Inventory, state	39.4 ± 8.3
***Adherence to MBI***	
completion rate^a^	33 (66%)
n. of attended sessions	6.9 ± 2.2

^a^proportion of participants who attended all 8 sessions at the end of the 8-week MBI

Among the 50 participants who initially enrolled in the online program, 33 successfully completed all 8 sessions, resulting in a completion rate of 66% (Table 1). The participants who did not complete the program were further categorized as follows: 8 attended seven sessions, 3 attended six sessions, 1 attended two sessions and 5 attended only one session. The withdrawals from the study were comprised of the 5 participants who attended just one session and an additional 3 participants who attended two, six or seven sessions. These withdrawals were primarily due to unforeseen medical issues (*n* = 2) or personal reasons (*n* = 1). Furthermore, one participant was unable to participate in the 6-month follow-up assessment due to intervening medical problems (Fig. 1).

### Longitudinal analysis

**Fig. 2:**
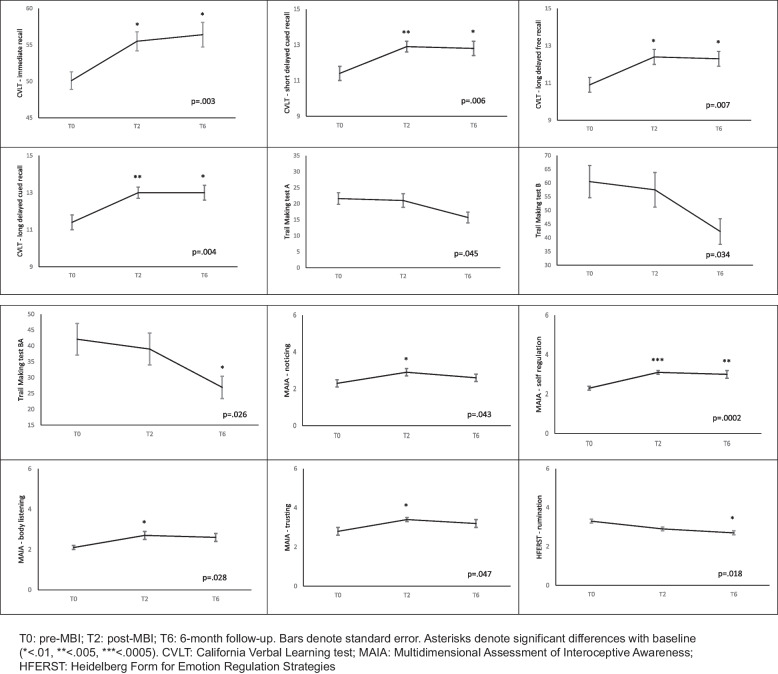
Estimated mean change in cognitive tests and psychological scales from pre to post MBI and 6-month follow-up

We observed significant improvements over time in several cognitive and psychological measures. Specifically, there were notable improvements in verbal memory, encompassing immediate, short-term and long-delayed cued and free recall from the CVLT (*p* ≤ 0.007). Furthermore, attention switching and executive functions, as assessed by the TMT versions A, B and BA, displayed significant improvements with *p*-values less than *p* < 0.05 (Fig. 2), Interoceptive awareness, as measured by the MAIA, exhibited significant enhancements in several dimensions, including noticing, self-regulation, body listening, and trusting dimensions, with a *p*-value of 0.0002 for self-regulation and *p*-values less than < 0.05 for the other dimensions. Additionally, rumination as evaluated using the HFERST showed a significant improvement (*p* = 0.018) (Fig. 2). For a visual representation of the raw data refer to Supplementary Figure 1 [see Supplementary figures]. There were no significant time effects for the other measures, as demonstrated in Supplementary Figure 2 [see Supplementary figures].

Post-hoc comparisons indicated that significant differences were observed between the pre-MBI and post-MBI assessments, as well as between the pre-MBI and T6 assessments. These differences were observed for the CVLT subscales and the MAIA self-regulation dimension. For the remaining three MAIA dimensions (noticing, body listening and trusting) significant differences were detected between the pre-MBI and post-MBI assessments. Additionally, significant differences were found for the TMTBA and the rumination measure between the pre-MBI and T6 assessments, as depicted in Fig. 2.

**Fig. 3:**
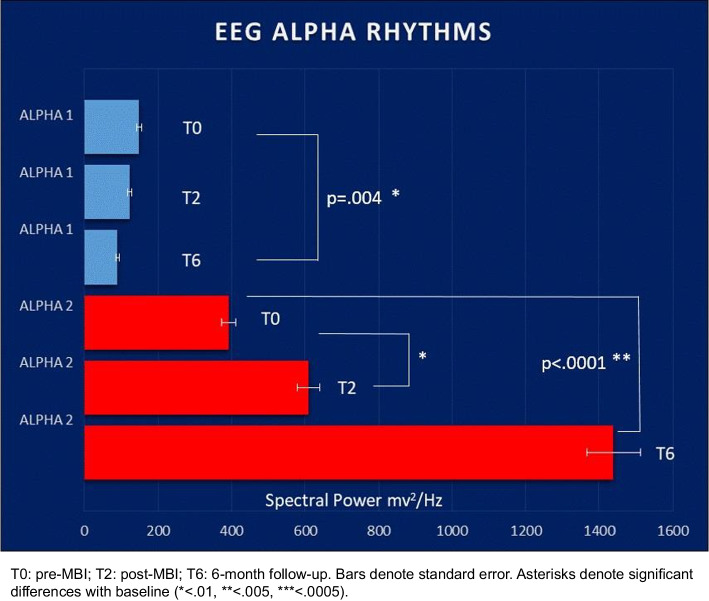
Mean change in EEG variables from pre to post MBI and 6-month follow-up

The EEG alpha1 power significantly decreased (*p* = 0.004), while alpha2 power significantly increased (*p* < 0.0001) from the pre-MBI to both the post-MBI and T6 assessments. Post-hoc comparisons revealed significant differences between the pre-MBI and T6 assessments for alpha1 and between the pre-to-post MBI assessments, as well as between the pre-MBI-to-T6 assessments for alpha2 (Fig. 3).

Table 2 provides information on the effect sizes of the significant changes observed in the study. The effect size was medium for immediate verbal memory (Ω^2^ = 0.075) and small to medium for delayed verbal memory (Ω^2^ between 0.061 and 0.068). It was small for attention switching and executive functions (Ω^2^ between 0.032 and 0.040), medium to large for the self-regulation dimension of interoceptive awareness (Ω^2^ = 0.12) and small for the other three dimensions (Ω^2^ between 0.033 and 0.042). The effect size for rumination was small (Ω^2^ = 0.048), while for EEG variables, it ranged from medium to very large for EEG variables (Ω^2^ 0.071 and 0.257).

**Table 2:** Estimated mean change and effect size of change in cognitive tests, psychological scales and EEG variables

	**T0**	**T2**	**T6**	**Ω**^**2**^
*Verbal memory*				
CVLT, immediate recall	50.1 + 1.2	55.5 + 1.3	56.4 + 1.7	0.075
CVLT, short delay cued recall	11.4 + .4	12.9 + .3	12.8 + .4	0.062
CVLT, long delay free recall	10.9 + .4	12.4 + .4	12.3 + .4	0.061
CVLT, long delay cued recall	11.4 + .4	13.0 + .3	13.0 + .4	0.068
*Attention switching and executive functions*				
Trail Making test A	21.6 + 1.8	21.0 + 2.1	15.7 + 1.7	0.032
Trail Making test B	60.5 + 5.9	57.5 + 6.3	42.3 + 4.7	0.036
Trail Making test BA	42.1 + 5.0	39.0 + 5.0	26.9 + 5.3	0.040
*Interoceptive awareness*				
MAIA, noticing	2.3 + .2	2.9 + .2	2.6 + .2	0.035
MAIA, self-regulation	2.3 + .1	3.1 + .1	3.0 + .2	0.12
MAIA, body listening	2.1 + .1	2.7 + .2	2.6 + .2	0.042
MAIA, trusting	2.8 + .2	3.4 + .1	3.2 + .2	0.033
*Emotion regulation strategies*				
HFESRT, rumination	3.3 + .1	2.9 + .1	2.7 + .1	0.048
*EEG variables*				
Alpha 1	148.4 + 8.7	122.6 + 9.6	89.0 + 15.7	0.071
Alpha 2	391.4 + 39.2	608.8 + 56.2	1439.4 + 155.6	0.257

*CVLT* California Verbal Learning test, *MAIA* Multidimensional Assessment of Interoceptive Awareness, *HFERST* Heidelberg Form for Emotion Regulation Strategies

### Correlation analysis

**Fig. 4:**
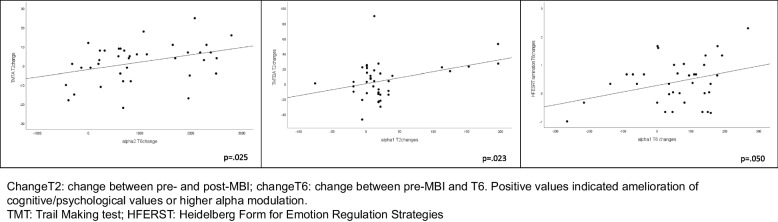
Significant correlations between cognitive or psychological changes and EEG changes

Figure 4 shows the significant correlations between different variables in the study. Specifically, T2change in TMTA was found to significantly correlate with T6change in alpha2 (*r* = 0.359, *p* = 0.025). Additionally, T2change in TMTBA exhibited a significant correlation with T2change in alpha1 (*r* = 0.363, *p* = 0.023), and T6change in rumination showed a marginally significant correlation with T6change in alpha1 (*r* = 0.330, *p* = 0.050). No other significant correlations were observed (data not shown).

## Discussion

This study investigated the impact of a live web-based MBI on the cognitive, psychological, and physiological aspects of healthy older adults during the COVID-19 pandemic. The findings indicate that participants improved in various domains, including verbal memory, attention switching and executive functions, interoceptive awareness, and rumination both pre-to-post MBI and at T6. Notably, the most significant changes, with medium effect sizes, were observed in immediate verbal memory (measured by CVLT) and self-regulation in interoceptive awareness (measured by MAIA), and these improvements were sustained at T6. Furthermore, the study revealed changes in EEG alpha1 and alpha2 activity modulation, which correlated with improvements in attention switching, executive function and rumination.

The observation of improved cognitive functions following a web videoconferencing MBI in our study represents a novel finding. Previous research primarily concentrated on the psychological impact of Internet-delivered MBIs, with a predominant focus on self-help courses [23, 66]. To the best of our knowledge, the study most closely resembling ours was conducted by Wang and colleagues [67], who explored cognitive changes resulting from a 6-week mindfulness training program delivered through a combination of face-to-face and videoconference modalities, involving 32 young adults. The findings align with our results, particularly with regards to the improvements observed in immediate recall (as measured with CVLT) post-MBI and at the 3-week follow-up, as well as enhanced attention switching (TMTA and TMTB) at the 3-week follow-up. Additionally, they demonstrated enhancements in executive functions based on performance in a maze task [67]. The computation of effect sizes in our study further supports these observations, indicating small to medium effects in these cognitive domains. In contrast, another study involving 53 older adults who underwent a brief—4-week—online mindfulness training via videoconferencing during the COVID-19 pandemic did not show positive effects on sustained attention [68].

The observed beneficial effect on attention and executive functions are consistent with existing literature and the theoretical models that describe the early cognitive mechanisms involved in mindfulness training. Traditional face-to-face MBIs have shown that attention switching and executive control are trained to redirect attention to the present moment when it wanders and to inhibit cognitive processes unrelated to the chosen focus of concentration [69]. A recent meta-analysis investigating the impact of MBIs on cognitive function in adults confirmed that MBIs exhibited superiority over comparators in enhancing executive function and working memory, albeit with a small effect size [15]. Significantly, our study uncovered a positive influence of MBI not only on immediate and short-term recall but also on long-term verbal episodic memory among older novice mindfulness practitioners. This novel observation enriches our understanding of mindfulness's potential to enhance memory functions in this demographic. Plausible mechanisms underlying mindfulness’s positive effects on episodic memory encompass increased attention, decreased cognitive interference [70] or improved encoding capabilities [71].

In terms of psychological outcomes, we did not observe a significant effect of MBI on self-reported mindfulness skills, which contrasts with the typical findings in the literature. However, our participants had higher baseline scores on the Five Facet Mindfulness Scale scores compared to similar populations [72]. Consequently, we hypothesize that there was limited room for improvement in our sample due to a potential floor effect. On the other hand, our study did reveal a significant impact of MBI on interoceptive awareness (IA). Specifically, we observed improvements in dimensions related to the awareness of bodily sensations (noticing), the integration of mind–body awarenes (self-regulation, body listening) and a greater sense of safety and trust in one's body (trusting), The effect size for self-regulation was medium.

The significance of IA plays in emotional experience, self-regulation, decision making, motivation, and overall well-being and mental health has gained increasing recognition [73, 74]. Otherwise, research has shown that IA tends to decline with age and this decline has been linked to age-related cognitive and socioemotional changes [75]. Mindfulness practices (e.g., breathing meditation, body scan, mindful walking) focus on anchoring one's attention to interoceptive signals in an open, non-judgmental and accepting manner [76]. In line with this, previous studies have reported positive effects of MBIs on various dimensions of MAIA in middle-aged adults [77, 78], adults with posttraumatic stress symptoms [79], and patients dealing with chronic pain and depression [80]. Our findings in older adults underscore the importance of considering IA as an outcome measure in MBIs for this age group. Further studies are warranted to evaluate changes in IA as a potential mechanism underlying the effects of common mindfulness interventions, as previously suggested [76].

Rumination involves the repetitive contemplation of the causes, consequences, and symptoms of negative affect, and it is regarded as a dysfunctional cognitive strategy for regulating emotions [81]. Research has consistently shown that rumination is linked to an inflexible cognitive style [82], and higher levels of rumination have been negatively correlated with attentional control [83]. Meditation practice can reduce rumination by redirecting attention away from ruminative thoughts and towards the present moment, while also fostering increased self-compassion [84]. A meta-analysis focusing on the impact of MBI on ruminative thoughts in patients with depression reported a moderate effect [85], and this effect has also been observed in older adults with depression [86]. Our discovery of reduced rumination following the MBI contributes to the existing body of literature and offers novel evidence into the context of healthy older adults.

In our EEG findings, we observed an increase in alpha2 power, often referred to as synchronization, and a decrease in alpha1 power, referred to as desynchronization. Alpha2 activity is typically considered an idling rhythm that appears in the cortex when there is no external stimulus. In contrast, alpha1 activity is associated with task that require internalized attention and alertness [35]. Drawing from the functional connection between EEG and functional magnetic resonance imaging signals [87–89], we can establish a relationship between alpha2 and the default mode network, which is the resting state network associated to spontaneous stimulus-independent thoughts. In contrast, alpha1 appears to be associated with the task-positive network, which is connected to higher cognitive functions, such as attention.

Given the existing evidence that mindfulness practice reduces activity in the default mode network due to decreased self-referential thinking and mind wandering [90], and simultaneously activates the task-positive brain network due to an improved ability to sustain attention during task performance [91], we posit that the observed increase in alpha2 activity and decrease in alpha1 activity could represent the corresponding physiological correlates of these cognitive changes. Synchronized oscillations in the alpha2 band may mirror a state of relaxed alertness following MBI. Desynchronized oscillations in the alpha1 band may indicate enhanced attentive skills, leading to a selective suppression of alpha activity across various sub-bands [31]. Supporting this hypothesis, we observed that the modulation of alpha activity correlated with improved attention and executive functions as well as reduced ruminations. The lack of a significative modulation in the upper alpha band (i.e., alpha3) following MBI aligns with the idea that alpha3 is selectively responsive to semantic memory demands [31] and that mindfulness practice may not have a specific impact on this form of memory [70]. In the literature, only a few studies have explored the neural correlates of meditative states using alpha oscillations defined in narrow frequency bands [92–95]. However, these studies differ significantly from ours in terms of target population (i.e., experienced vs novice practitioners) and the types of meditation employed (non-MBIs, i.e., Qigong). Due to these variations, a direct comparison with our findings is limited.

One primary limitation of this study is the lack of an active control group, which makes it difficult to rule out the possibility that the observed benefits are due to non-specific factors, such as participation in a research group or expectancy effects [96]. Nevertheless, the significant correlations between cognitive/psychological measures and EEG variables provide some evidence that the clinical benefit we observed may have a neurobiological basis, reducing the plausibility of confounding by non-specific factors. A second important limitation to consider is the small sample size, which emphasizes the need for confirmation of our preliminary results in larger studies. This limitation is intrinsic to the exploratory design of the study but is partially mitigated by the comprehensive collection of cognitive and psychological variables in the same group of subjects. This dataset serves as a foundation for the methods and procedures to be employed in larger studies.

## Conclusions

The scarcity of studies examining the effects of web-based mindfulness interventions in older adults is a notable gap in the current research landscape. This area of study holds significant importance, especially in the context of the present times where traditional in-person group activities may not always be feasible or safe for older adults.

Our findings contribute new insights to this field of study, indicating that web videoconferencing MBI in older adults lead to improved cognitive and psychological measures with small to medium effect sizes. These positive effects are sustained over a 6-month follow-up period. Furthermore, our results reveal correlations between measures of cognitive and psychological improvement and neural activity in specific brain EEG rhythms, suggesting that the observed clinical benefits may have a neurobiological basis. Further controlled studies are essential to confirm these preliminary findings.

### Future directions

The growing public and research interest in online MBIs is evident [17, 23], and it is reasonable to anticipate an increasing adoption of these interventions in both clinical and non-clinical settings in the near future. Nevertheless, in our opinion, there are several priority issues that need to be addressed before online MBIs can be proposed as add-on or alternative interventions to traditional, in-person MBIs. A significant challenge lies in the heterogeneity of these interventions. Currently, there are nearly 200 mindfulness apps [22] and over 80 web-based interventions [23], yet there is a lack of a standardized curriculum, analogous to the well-established MBSR or MBCT programs in traditional MBIs. Another major limitation in the field of online MBIs is the lack of high-quality randomized controlled trials demonstrating that online and traditional MBIs equally and positively affect mental health. While research in this area is still in its early stages, a few comparative studies have suggested that online MBIs may be equally effective [97–99]. Notably, these studies compared traditional MBIs with online interventions delivered by videoconferencing [97] or through web-based programs that included interaction with a teacher [98, 99]. This implies that the role of a skilled mindfulness instructor is pivotal in determining the overall benefit of online MBIs. A guided approach may be particularly suitable for novice older adults, who could potentially face greater challenges in comprehending written instructions on mindfulness practice or in maintaining their focus during online individual sessions, especially when compared to young individuals. To the best of our knowledge, there are no studies available that directly compare mindfulness apps to in-person interventions. This remains an important area for future research.

The limitations of our study suggest additional directions for future research. A critical issue is the use of an active control to test the efficacy of MBIs. While there is substantial evidence that MBIs improve outcomes compared to inactive control groups (i.e., waiting list) [100], evidence also points out that effect sizes are smaller when compared to active controls, for both in-person [2] and online [23] MBIs. Therefore, randomised trials adopting well-designed and validated active control interventions are warranted. Another key issue pertained to health literacy, i.e., the ability of an individual to obtain and translate knowledge and information to maintain and improve health [101]. It is noteworthy that our study sample was in partly drawn from individuals who had participated in previous research studies and expressed interest in future research. This may have introduced a selection bias, potentially resulting in an overrepresentation of more educated participants with high literacy, and limiting the generalizability of the results. On the other hand, by emphazing individuals' intrinsic capacities to attain health and fully function, the delivery of mindfulness practice through information and communication technologies can play a central role in advancing person-centered healthcare and enhancing health literacy in older adults. This is an under-research topic [102] that deserves further exploration in larger studies representative of the general population.

### Supplementary Information

**Additional file 1: Supplementary Table 1.** Description of practices included in each mindfulness-based intervention session and engaged at home.** Supplementary table 2.** Psychological features of older adults attending mindfulness-based intervention.** Supplementary Table 3.** Cognitive and personality features of older adults attending mindfulness-based intervention.** Supplementary table 4.** Psychological and clinical features of older adults attending mindfulness-based intervention.

**Additional file 2: Supplementary Figure 1.** Spaghetti-plot of raw longitudinal data on cognitive tests (panel A), psychological scales (panel B), and EEG variables (panel C) from pre to post MBI and 6-month follow-up. The superimposed red lines indicate estimated means.** Supplementary Figure 2.** Estimated mean change in cognitive tests, psychological scales, and EEG variables from pre to post MBI and 6-month follow-up. Estimated mean and standard error; *p*-value adjusted for age from mixed models.

## Data Availability

The dataset analyzed in this article is publicly available at the Mendeley data repository (https://data.mendeley.com/datasets/x3vb85t4xh/1).

## Abbreviations

MBIs: Mindfulness-based interventions
EEG: Electroencephalography
CVLT: California Verbal Learning Test
TMT: Trail Making Test
HFERST: Heidelberg Form for Emotion Regulation Strategies
MAIA: Multidimensional Assessment of Interoceptive Awareness
TF: Transition Frequency
IAF: Individual Alpha Frequency
Ω^2^: Omega Squared
IA: Interoceptive Awareness
