# Supplementary material for: Cognitive, psychological, and physiological effects of a web-based mindfulness intervention in older adults during the COVID-19 pandemic: an open study
Source: BMC Geriatr. 2024 Feb 14;24:151. doi: 10.1186/s12877-024-04766-z (PMC10865647; doi:10.1186/s12877-024-04766-z)
Supplement: Supplementary file 1 — Additional file 1: Supplementary Table 1. Description of practices included in each mindfulness-based intervention session and engaged at home. Supplementary table 2. Psychological features of older adults attending mindfulness-based intervention. Supplementary Table 3. Cognitive and personality features of older adults attending mindfulness-based intervention. Supplementary table 4. Psychological and clinical features of older adults attending mindfulness-based intervention. [file 12877_2024_4766_MOESM1_ESM.docx]

**SUPPLEMENTARY MATERIAL**

**FILE 1: SUPPLEMENTARY TABLES**

**Supplementary table 1** Description of practices included in each mindfulness-based intervention session and engaged at home

| **Session** | **Practices** | **Homework** |
| --- | --- | --- |
| ***1*** *Automatic pilot* | Raisin exercise, body scan (45 minutes) | Body-scan + mindful eating + mindful awareness of one routine activity |
| ***2*** *How does my mind work?* | Body scan, sitting meditation: mindful breathing (15 minutes); exercise: “Thoughts and emotions” | Body scan + sitting meditation: mindful breathing + diary of pleasant events + mindful awareness of one routine activity |
| ***3*** *Breath as an anchor point to the present moment* | sitting meditation: mindful breathing, awareness of pleasant events; gentle yoga | sitting meditation: mindful breathing + body scan or yoga + diary of unpleasant events + mindful awareness of one routine activity |
| ***4*** *Stress reaction cycle* | mindful walking, sitting meditation: mindful breathing and mindfulness of the whole body, awareness of unpleasant events and psychoeducation about the stress reaction cycle, 3 minutes breathing space | mindful walking + sitting meditation: mindful breathing and mindfulness of the whole body or yoga + three-minutes breathing space practice at predetermined moments + reactivity’s diary + mindful awareness of one routine activity |
| ***5*** *Managing stress and letting go* | sitting meditation: mindful breathing, mindfulness of the whole body and of sounds, recognizing aversion (reactivity’s diary) and psychoeducation on stress management | Yoga + mindful walking or sitting meditation: mindful breathing, mindfulness of the whole body and of sounds + three-minutes breathing space at predetermined moments and in challenging situations practices + mindful awareness of one routine activity |
| ***6*** *Thoughts are not facts* | Gentle Yoga, complete sitting meditation: mindful breathing, body, sounds, thoughts and open awareness, three-minutes breathing space, exercise: thoughts are not facts | Yoga + complete sitting meditation + three-minutes breathing space practice at predetermined moments and in challenging situations practices + mindful awareness of one routine activity |
| ***7*** *Dealing with difficulties* | Complete sitting meditation with focus on a difficult situation (guided imagery), how can I best take care of myself, forgiveness meditation | 45 minutes of practices (free program), three-minutes breathing space practice at predetermined moments and in challenging situations practices, informal practices |
| ***8*** *Maintaining discipline* | Body scan (15 minutes), yoga, complete sitting meditation | Establish one’s mindfulness meditation practice for daily life |
| *Booster session* | One half day of mindful practice and Lecture (acceptance and change), practices, mountain meditation, obstacles to practice, poetry | Continue one own’s mindfulness meditation practice |

**Supplementary table 2** Psychological features of older adults attending mindfulness-based intervention

| ***Psychological scale (Italian version)*** | ***Cronbach alpha*** |
| --- | --- |
| **Five Facet Mindfulness scale** *(Didonna 2012)* | |
| observing | 0.80 |
| describing | 0.88 |
| acting with awareness | 0.86 |
| non-judging of inner experience | 0.85 |
| non-reactivity to inner experience | 0.66 |
| **Multidimensional Assessment of Interoceptive Awareness** *(Calì 2015)* | |
| noticing | 0.68 |
| not distracting | 0.53 |
| not worrying | 0.59 |
| attention regulation | 0.75 |
| emotional awareness | 0.79 |
| self-regulation | 0.75 |
| body listening | 0.74 |
| trusting | 0.80 |
| **Penn State Worry Questionnaire** *(Morani 1999)* | 0.85 |
| **Warwick-Edinburgh Mental Well-being scale** *(Gremigni 2011)* | 0.86 |
| **Emotion Regulation Questionnaire** *(Balzarotti 2010)* |  |
| cognitive reappraisal | 0.84 |
| expressive suppression | 0.72 |
| **Heidelberg Form for Emotion Regulation Strategies** *(Iani 2019)* | |
| rumination | 0.76 |
| reappraisal | 0.75 |
| acceptance | 0.77 |
| problem-solving | 0.90 |
| suppression of emotional expression | 0.86 |
| suppression of emotional experience | 0.75 |
| avoidance | 0.70 |
| activities and social support | 0.60 |
| distraction | 0.56 |
| **Pittsburgh Sleep Quality index** *(Curcio 2013)* | 0.84 |
| **Multifactorial Memory Questionnaire** *(Raimo 2016)* | |
| MMQ satisfaction | 0.90 |
| MMQ ability | 0.91 |
| MMQ strategy | 0.87 |
| **NEO Personality Inventory-3** *(Fossati 2014)* |  |
| Neuroticism | 0.90 |
| Extraversion | 0.88 |
| Openness | 0.88 |
| Agreeableness | 0.81 |
| Conscientiousness | 0.91 |

Didonna F. Assessing mindfulness skills: A validation study of the Italian version of the Five Facet Mindfulness Questionnaire. Psicoterapia Cognitiva e Comportamentale. 2012;18:261-84.

Calì G, Ambrosini E, Picconi L, Mehling WE and Committeri G. Investigating the relationship between interoceptive accuracy, interoceptive awareness, and emotional susceptibility. Front. Psychol. 2015;6:1202.

Morani S, Pricci D, Sanavio E. «Penn State Worry Questionnaire» e «Worry Domains Questionnaire»: Presentazione delle versioni italiane ed analisi della fedeltà. Psicoterapia Cognitiva e Comportamentale. 1999;5:195-209.

Gremigni P, Stewart-Brown S. Una misura del benessere mentale: validazione italiana della Warwick-Edinburgh Mental Well-Being Scale (WEMWBS). Giornale Italiano di Psicologia. 2011;2:543-63.

Balzarotti S, John O, Gross J. An Italian Adaptation of the Emotion Regulation Questionnaire. European Journal of Psychological Assessment. 2010;26:61-7.

Iani L, Lauriola M, Chiesa A, Cafaro V. Associations between mindfulness and emotion regulation: The key role of describing and nonreactivity. Mindfulness. 2019;10:366-75.

Curcio G, Tempesta D, Scarlata S, Marzano C, Moroni F, Rossini PM, Ferrara M, De Gennaro L. Validity of the Italian Version of the Pittsburgh Sleep Quality Index (PSQI). Neurol Sci. 2013;34:511–9.

Raimo S, Trojano L, Siciliano M, Cuoco S, D’Iorio A, Santangelo F, Abbamonte L, Grossi D, Santangelo G. Psychometric properties of the Italian version of the multifactorial memory questionnaire for adults and the elderly. Neurol Sci. 2016;37:681–91.

Fossati L, Ciancaleoni M. NEO-PI-3 (adattamento italiano). 2014. Hogrefe Editore, Firenze.

**Supplementary table 3** Cognitive and personality features of older adults attending mindfulness-based intervention

|  | **N=50** |
| --- | --- |
| ***Cognitive features*** |  |
| *Verbal memory* *(California Verbal Learning test)* |  |
| immediate recall | 50.1+8.6 |
| short delay free recall | 10.6+3.2 |
| short delay cued recall | 11.4+2.7 |
| long delay free recall | 10.9+2.6 |
| long delay cued recall | 11.4+2.6 |
| *Attention and processing speed* |  |
| Attentional matrices | 42.5+5.9 |
| Trail Making test A | 21.7+12.6 |
| Stroop test, time | 11.3+7.9 |
| *Executive functions* |  |
| Trail Making test B | 60.2+41.0 |
| Stroop test, error interference score | -.14+1.07 |
| Wisconsin Card Sorting test, global score | 43.8+34.4 |
| Wisconsin Card Sorting test, errors | 11.6+11.9 |
| Wisconsin Card Sorting test, perseverations | 12.5+10.7 |
| ***Personality*** *(NEO Personality Inventory)* |  |
| Neuroticism | 45.5+8.5 |
| Extraversion | 46.5+7.6 |
| Openness | 49.1+8.3 |
| Agreeableness | 59.4+8.4 |
| Conscientiousness | 54.2+7.9 |

WCST: 4 missing; NEO-PI: 9 missing

**Supplementary table 4** Psychological and clinical features of older adults attending mindfulness-based intervention

|  | **N=50** |
| --- | --- |
| ***Psychological features*** |  |
| ***Dispositional mindfulness*** *(Five Facet Mindfulness scale)* |  |
| observing | 25.6+4.2 |
| describing | 26.7+4.9 |
| acting with awareness | 28.1+5.2 |
| non-judging of inner experience | 27.9+5.6 |
| non-reactivity to inner experience | 20.5+3.7 |
| ***Interoceptive awareness*** *(Multidimensional Assessment of Interoceptive Awareness)* |  |
| noticing | 2.2+1.2 |
| not distracting | 2.1+.6 |
| not worrying | 2.6+.9 |
| attention regulation | 2.3+1.0 |
| emotional awareness | 2.9+1.2 |
| self-regulation | 2.3+1.0 |
| body listening | 2.1+1.0 |
| trusting | 2.8+1.3 |
| ***Chronic worry*** |  |
| Penn State Worry Questionnaire | 48.1+11.0 |
| ***Psychological well-being*** |  |
| Warwick-Edinburgh Mental Well-being scale | 50.8+5.2 |
| ***Emotion regulation strategies*** |  |
| *Emotion Regulation Questionnaire* |  |
| cognitive reappraisal | 28.9+5.9 |
| expressive suppression | 14.5+4.8 |
| *Heidelberg Form for Emotion Regulation Strategies* |  |
| rumination | 3.3+.9 |
| reappraisal | 3.1+.9 |
| acceptance | 3.5+.9 |
| problem-solving | 3.9+.7 |
| suppression of emotional expression | 2.9+.9 |
| suppression of emotional experience | 2.7+.8 |
| avoidance | 2.9+.8 |
| activities and social support | 2.8+.8 |
| distraction | 2.0+.8 |
| ***Clinical features*** |  |
| ***Sleep quality*** |  |
| Pittsburgh Sleep Quality index | 6.6+3.3 |
| **Memory concerns** *(Multifactorial Memory Questionnaire)* |  |
| MMQ satisfaction | 37.7+9.0 |
| MMQ ability | 49.3+10.7 |
| MMQ strategy | 29.3+11.0 |
