# Supplementary material for: Cognitive, psychological, and physiological effects of a web-based mindfulness intervention in older adults during the COVID-19 pandemic: an open study
Source: BMC Geriatr. 2024 Feb 14;24:151. doi: 10.1186/s12877-024-04766-z (PMC10865647; doi:10.1186/s12877-024-04766-z)
Supplement: Supplementary file 2 — Additional file 2: Supplementary Figure 1. Spaghetti-plot of raw longitudinal data on cognitive tests (panel A), psychological scales (panel B), and EEG variables (panel C) from pre to post MBI and 6-month follow-up. The superimposed red lines indicate estimated means. Supplementary Figure 2. Estimated mean change in cognitive tests, psychological scales, and EEG variables from pre to post MBI and 6-month follow-up. Estimated mean and standard error; p-value adjusted for age from mixed models. [file 12877_2024_4766_MOESM2_ESM.docx]

**SUPPLEMENTARY MATERIAL**

**FILE 2: SUPPLEMENTARY FIGURES**

**Supplementary figure 1** Spaghetti-plot of raw longitudinal data on cognitive tests (panel A), psychological scales (panel B), and EEG variables (panel C) from pre to post MBI and 6-month follow-up. The superimposed red lines indicate estimated means

| Panel A – Cognitive tests | | |
| --- | --- | --- |
|  |  |  |
|  |  |  |
|  |  |  |
| Panel B – Psychological scales | | |
|  |  |  |
|  |  |  |
| Panel C – EEG variables | | |
|  |  |  |

CVLT: California Verbal Learning test MAIA: Multidimensional Assessment of Interoceptive Awareness; HFERST: Heidelberg Form for Emotion Regulation Strategies.

**Supplementary figure 2** Estimated mean change in cognitive tests, psychological scales, and EEG variables from pre to post MBI and 6-month follow-up. Estimated mean and standard error; p-value adjusted for age from mixed models

|   p=.068 |   p=.37 |   p=.30 |   p=.18 |
| --- | --- | --- | --- |
|   p=.15 |   p=.18 |   p=.086 |   p=.44 |
|   p=.71 |   p=.52 |   p=.063 |   p=.68 |
|   p=.57 |   p=.73 |   p=.18 |   p=.085 |
|   p=.47 |   p=.18 |   p=.42 |   p=.27 |
|   p=.76 |   p=.74 |   p=.99 |   p=.55 |
|   p=.81 |   p=.67 |   p=.34 |   p=.39 |
|   p=.15 |   p=.13 |   p=.49 |   p=.14 |
|   p=.62 |   p=.68 |   p=.10 |  |

CVLT: California Verbal Learning test; WCST: Wisconsin Card Sorting test; STAI: State-Trait Anxiety Inventory; MAIA: Multidimensional Assessment of Interoceptive Awareness; ERQ: Emotion Regulation Questionnaire; HFERST: Heidelberg Form for Emotion Regulation Strategies; MMQ: Multifactorial Memory Questionnaire.
